# Measurement properties of the EQ-5D-5L compared to EQ-5D-3L in the Thai diabetes patients

**DOI:** 10.1186/s12955-014-0203-3

**Published:** 2015-02-06

**Authors:** Juntana Pattanaphesaj, Montarat Thavorncharoensap

**Affiliations:** Social and Administrative Pharmacy Excellence Research Unit (SAPER Unit), Department of Pharmacy, Faculty of Pharmacy, Mahidol University, 447 Sri-Ayuthaya Rd., Rajathevi, Bangkok, 10400 Thailand; Health Intervention and Technology Assessment Program (HITAP), 6th Floor, 6th Building, Department of Health, Ministry of Public Health, Tiwanon Rd., Muang, Nonthaburi 11000 Thailand

**Keywords:** Diabetic, EQ-5D-3L, EQ-5D-5L, Health-related quality of life, Measurement properties, Psychometrics

## Abstract

**Background:**

The EQ-5D is a health-related quality of life instrument which provides a simple descriptive health profile and a single index value for health status. The latest version, the EQ-5D-5L, has been translated into more than one hundred languages worldwide - including Thai. This study aims to assess the measurement properties of the Thai version of the EQ-5D-5L (the 5L) compared to the EQ-5D-3L (the 3L).

**Methods:**

A total of 117 diabetes patients treated with insulin completed a questionnaire including the 3L and the 5L. The 3L and 5L were compared in terms of distribution, ceiling, convergent validity, discriminative power, test-retest reliability, feasibility, and patient preference. Convergent validity was tested by assessing the relationship between each dimension of the EQ-5D and SF-36v2 using Spearman’s rank-order correlation. Discriminative power was determined by the Shannon index (*H* ′) and Shannon’s Evenness index (*J* ′). The test-retest reliability was assessed by examining the intraclass correlation coefficient (ICC) and Cohen’s weighted kappa coefficient.

**Results:**

No inconsistent response was found. The 5L trended towards a slightly lower ceiling compared with the 3L (33% versus 29%). Regarding redistribution, 69% to 100% of the patients answering level 2 with the 3L version redistributed their responses to level 2 with the 5L version while about 9% to 22% redistributed their responses to level 3 with the 5L version. The Shannon index (*H* ′) improved with the 5L while the Shannon's Evenness index (*J* ′) reduced slightly. Convergent validity and test-retest reliability was confirmed for both 3L and 5L.

**Conclusions:**

Evidence supported the convergent validity and test-retest reliability of both the 3L and 5L in diabetes patients. However, the 5L is more promising compared to the 3L in terms of a lower ceiling, more discriminatory power, and higher preference by the respondents. Thus, the 5L should be recommended as a preferred health-related quality of life measure in Thailand.

**Electronic supplementary material:**

The online version of this article (doi:10.1186/s12955-014-0203-3) contains supplementary material, which is available to authorized users.

## Background

The EQ-5D - a widely used generic instrument for describing and valuing health outcomes in clinical and economic evaluations - was originally developed in the 1980s [1,2]. Due to its simplicity and brevity, it imposes minimal respondent burden and can be administered using a variety of modalities including self-completion. Many health technology assessment (HTA) organizations including the National Institution for Clinical Excellence (NICE) [3], the US panel on Cost-effectiveness in Health and Medicine [4], and the Thai national guideline of HTA [5] have recommended the EQ-5D as the preferred method for assessing the utility for health technology assessment.

The EQ-5D comprises 2 parts: a simple descriptive profile that can be converted into a single summary index (the EQ-5D index), and a visual analog scale (VAS). At present, the first version of the EQ-5D - known as EQ-5D-3L version (hereafter “the 3L”) - has now been translated into more than 140 languages [6]. The 3L descriptive system is composed of five dimensions: mobility; self-care; usual activities; pain/discomfort; and anxiety/depression. Each dimension has three levels of impairment, namely no problems (level 1), some/moderate problems (level 2), and extreme problems (level 3). The descriptive response from the EQ-5D can be converted into an index score which is useful for clinical and economic evaluations [2]. For the VAS, a respondent will be asked to rate their health on a 20-centimeter vertical scale. The scale ranges from 0 to 100, where 0 means the worst possible health that the respondent can imagine and 100 indicates the best possible health in the respondent’s viewpoint.

Since the 3L is limited to three levels of response categories, a substantial ceiling effect was observed [7-12]. In addition, it has limitations in measuring small changes, especially in mild conditions [13-16]. Previous studies also found that the 3L appeared to be less sensitive when compared to the SF-12 or SF-36 [7,8]. In response to the problems previously mentioned, the 5-level of EQ-5D (EQ-5D-5L, hereafter “the 5L”) was developed by a task force within the EuroQol group [13,14]. This version includes five levels of impairment in each of the existing five EQ-5D dimensions. At present, the 5L has now been translated into more than 113 languages [17]. Several studies [15,16,18-24] examining the measurement properties of the 5L have found that it is a valid and reliable instrument. When comparing the 5L with the 3L, it was found that the 5L had a lower ceiling effect [16,18-21,23,24] and greater discriminative power with the potential to better detect the differences between groups [15,16,18,20,21,24]. In addition, it showed better face validity [13,15,25] and test-retest reliability [18,21,23].

Previous studies were conducted in several countries to evaluate the measurement properties of the 3L compared to those of the 5L [15,16,18-24]. However, there is a substantial need to assess the measurement properties of the 5L in different populations and patients. The Thai version of EQ-5D-5L has been available since 2013 but there has been no assessment of its measurement properties in Thailand to our knowledge. Therefore, this study aims to examine this issue and to assess the measurement properties of the 5L in comparison with the 3L among diabetes mellitus patients treated with insulin. The measurement properties will be assessed in terms of distribution; redistribution; ceiling; convergent validity; discriminative power; test-retest reliability; feasibility; and patient preference.

## Methods

### Subjects and settings

A convenience sample of patients with diabetes mellitus - who received treatment at the outpatient department at Ramathibodi Hospital, Thailand during 7 January and 31 March 2013 - was invited to participate in this study. Patients were eligible if they met the following criteria: aged ≥ 12 years, required regular insulin treatment, and had no complications as determined by the nurse. Pregnant women and disabled persons were excluded from this study.

### Procedure and instruments

The questionnaire consisted of 4 parts: 1) one page of the Thai version of the 3L and 5L response scale; 2) the EQ-VAS; 3) two preference questions; and 4) the short-form 36 health survey version 2 (SF-36v2) in Thai. The permission to use the official Thai version of the 3L, 5L, and SF-36v2 was granted by the authoritarians before beginning the data collection process.

The single page of the 3L and 5L response scale contained the 5L version on the left column and the 3L version on the right column. Similar to previous studies [15,18,20], respondents were asked to complete the 5L first, followed by the 3L in order to avoid the tendency to not choose levels 2 and 4 - the “in-between” options - when the 3L was completed first. The index value of the 5L was obtained from an interim mapping generated by the EuroQol group [26] as the valuation study of the 5L in Thailand has not yet been completed. The 3L index value was calculated using the Thai value sets studied by Tongsiri et al. [27].

The preference questions comprised 2 items: 1) Which response scale is easier to use? (the 3L or the 5L or indifferent); and 2) Which response scale best describes your health? [15].

The convergent validity of the 5L and 3L were evaluated by comparing them with the SF-36 as it is a widely-used generic health survey in clinical research and has demonstrated validity among the Thai population [28-30]. The SF-36 contains 8 dimensions, i.e. physical functioning; role limitation due to physical problems; bodily pain; general health perceptions; social functioning; vitality; role limitations due to emotional problems; and general mental health [31]. Since a weighted Likert scale is used as the scoring system, the items for each dimension are summed to provide a score which is then linearly transformed into a value from 0 – 100 (100 indicating the best health level).

This study was approved by the Mahidol University Institutional Review Board (MU-IRB), Thailand and the Institute for the Development of Human Research Protections (IHRP), Ministry of Public Health, Thailand. All participants provided written informed consent and all instruments were self-administered. After completing the questionnaire, the respondents received 3.25 USD for compensation (1 USD = 30.73 Baht). All respondents were also asked to complete a second set of questionnaires after 2 weeks and to return it by mail; the set consisted of one page of the Thai 3L and 5L response scale and the EQ-VAS. If the second questionnaire did not reach the researcher within 3 days after due date, phone call or short message was made to remind the respondent. The second questionnaires which reached to the researcher later than 21 days were excluded from the analysis.

### Statistical analyses

The distribution of the 3L and 5L responses was demonstrated in terms of percentage of each level reported. The redistribution patterns of the responses from the 3L to 5L for each dimension were also reported in terms of percentage. Similar to previous studies [15,21], the response inconsistency and size were determined and are shown in Table 1. To determine the inconsistency, the response of the 3L was converted into the 5L (the 3L_5L_) as follows: 1 = 1, 2 = 3, and 3 = 5. Then, the size of inconsistency was calculated as |3L_5L_-5L|-1. A size of inconsistency of ≤ 0 indicated consistency, and thus only 7 pairs are considered as consistent responses.

**Table 1:** **Size of (in) consistent response**

**3L**	**5L**				
	**Level 1**	**Level 2**	**Level 3**	**Level 4**	**Level 5**
**level 1**	-1	0	1	2	3
**level 2**	1	0	-1	0	1
**level 3**	3	2	1	0	-1

Adapted from Janssen et al [16]. The size of inconsistency of ≤ 0 indicated consistency.

For the ceiling, the proportion of respondents reported ‘no problems’ for all five dimensions - the proportion of respondents scoring ‘11111’ [16] - was compared for the 3L and 5L. The percentage reduction from the 5L to 3L was calculated as follows: (Ceiling 3L – Ceiling 5L)/ Ceiling 5L. We hypothesized that the ceiling should be lower in the 5L compared with the 3L. Feasibility was assessed by calculating the number of missing values for the 5L and 3L.

Convergent validity was tested by assessing the relationship between each dimension of the 5L and SF-36v2 using Spearman’s rank-order correlation (Spearman’s rho). We hypothesized that each dimension in the 5L would be more highly correlated to related subscales than to other subscales in the SF-36 compared to the 3L. Specifically, we expect to see strong correlation between these pairs of subscales: mobility and physical functioning; pain and bodily pain; anxiety/depression and mental health. We also expected to identify moderate correlation between these pairs of subscales: self-care and physical functioning or role limitation due to physical problems; usual activity and role limitation due to physical problems. The EQ-5D’s responses were recoded to signify that higher scores presented better health statuses. The strength of correlation was determined as follows: absent (r < 0.20), weak association (0.2 ≤ r < 0.35), moderate (0.35 ≤ r < 0.50), and strong (r ≥ 0.50) [32]. Additionally, the relationship between VAS score and index value was reported using the Pearson’s correlation coefficient.

Discriminative power (or informativity) was determined by the Shannon index (*H* ′) and Shannon’s Evenness index (*J* ′). *H* ′ and *J* ′ are often used to reflect the discriminatory power of health state classification [15,16,18,21,33]. *H* ′ reflects the absolute information content. The higher the *H* ′, the more information is captured by the measure. On the other hand, *J* ′ expresses the relative informativity of a system or the evenness of a distribution regardless of the number of categories. In case of an even distribution - when all levels are filled with the same frequency - *J* ′ is equal to 1. When comparing the 5L to the 3L, we expect the *H* ′ of the 5L to be higher to reflect more discriminatory performance. On the other hand, the *J* ′ of the 5L might slightly decrease as the extra level might not be used equally.

The test-retest reliability of both EQ-5D index scores was evaluated using the intraclass correlation coefficient (ICC) and the reliability of each dimension was assessed with Cohen’s weighted kappa coefficient. According to Fleiss’s standards for the strength of agreement for kappa values [34], Cohen’s weighted kappa (k) was determined as follows: poor reproducibility (k < 0.4); good reproducibility (0.4 ≤ k < 0.75; excellent reproducibility (k ≥ 0.75). Regarding intra-rater reliability among each dimension at different times, the data set lacked variance since most respondents responded with level 1 for self-care. The weighted kappa coefficient could not be calculated, thus percentage agreement values was demonstrated also [35,36]. It was calculated as: (a + d)/N, where the values of a and d were obtained from a 2x2 table.

All data were analyzed using SPSS 19. Statistical significance was set a priori as p < 0.05.

## Results

### Characteristics of respondents

A total of 117 patients with diabetes mellitus who met the eligibility criteria were included. The characteristics of the respondents are shown in Table 2. The average age of the respondents was 45 years, with 62.4% being female. Sixty-four (54.7%) respondents had type 2 diabetes. The average diabetes duration of the sample was 9 years and the average BMI was 23.30. Of the 117 respondents who completed the first survey, 101 respondents (86%) returned the second questionnaire set by postal mail.

**Table 2:** **Demographic characteristic of respondents**

**Demographic characteristic**	**n (%)**
**Type of diabetes**	
Type 1	53 (45.3)
Type 2	64 (54.7)
**Gender**	
Male	44 (37.6)
Female	73 (62.4)
**Marital status**	
Single	58 (49.6)
Married	46 (39.3)
Widowed	9 (7.7)
Divorced/Separated	4 (3.4)
**Education**	
High school	51 (43.6)
Primary school	27 (23.1)
Bachelor’s degree	25 (21.4)
Diploma	10 (8.5)
Master’s degree or higher	4 (3.4)
**Occupation**	
Student	50 (42.7)
Government/state enterprise officer	20 (17.1)
Housewife	14 (12.0)
Business owner	11 (9.4)
Unskilled labor	7 (6.0)
Retired	6 (5.1)
Employee	4 (3.4)
Agriculture/fishery	2 (1.7)
Other	3 (2.6)
**Health insurance**	
Civil Servants Medical Benefits Scheme	58 (49.6)
Out of pocket	32 (27.4)
Universal coverage	20 (17.1)
Social security	7 (6.0)
	**Median (IQR)**
Age (years)	45.00 (40.0)
Diabetes duration (yr)	9.00 (8.50)
BMI (Kg/M^2^)	23.30 (7.37)
Household income per month (Baht)	30,000 (30,000)

The health state ‘11111’ was observed in 29.1% in the 5L and 33.3% for the 3L. The second-most frequent health state reported was ‘11121’ which was 14.5% in the 5L and 15.4% in the 3L. Finally, there were no missing values from both the 5L and the 3L, indicating good feasibility for both instruments.

### Distribution and ceiling

**Figure 1:**
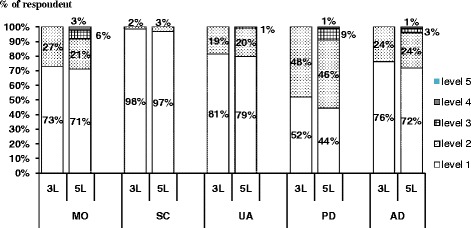
**Distribution across severity level of the 3L and 5L dimension.**

For all of the dimensions, most respondents reported no problems (level 1) for both the 3L (52-98%) and the 5L (44-97%), as shown in Figure 1. Among responses with health problems, it was clear that the 5L demonstrated better severity level distribution than the 3L except for self-care.

With regards to the ceiling, the 5L showed a slightly decreasing trend for no problem responses compared with the 3L. The percentage of patients reporting the health state ‘11111’ decreased from 33% in the 3L to 29% in the 5L. Nevertheless, no statistically significant difference was found. Self-care reached the highest ceiling (98% for the 3L, 97% for the 5L) and showed the smallest reduction in ceiling (1%) with the 5L. In contrast, pain/discomfort showed the smallest ceiling (52% for the 3L, 44% for the 5L) and also showed statistically significant reduction in ceiling with the 5L. No statistically significant reduction was found for the other dimensions.

### Redistribution

Among the answers of no problem (level 1) on the 3L, most of them (85-98%) remained the same (no problem) on the 5L while 2-15% redistributed to slight problems (level 2) on the 5L as shown in Table 3. The majority of the respondents who reported moderate problems (level 2) on the 3L indicated slight problems (level 2) on the 5L (69-100%), while 9-22% shifted to moderate problems (level 3) on the 5L. As such, redistribution occurred the least in self-care. The mean VAS score tended to be lower according to the severity level of the 5L. No inconsistent response was found in this study.

**Table 3:** **Redistribution pattern of response from 3L to 5L**

**Dimension**	**3L**	**5L**	**n (%)**	**Mean VAS**	Size of inconsistent response*
Mobility	1	1	83 (98%)	81.02	−1
		2	2 (2%)	85.00	0
	2	2	22 (69%)	72.38	0
		3	7 (22%)	71.43	−1
		4	3 (9%)	72.67	0
Self-care	1	1	113 (98%)	79.19	−1
		2	2 (2%)	70.00	0
	2	2	2 (100%)	60.00	0
Usual activities	1	1	93 (98%)	80.82	−1
		2	2 (2%)	80.00	0
	2	2	20 (91%)	71.85	0
		3	2 (9%)	50.00	−1
Pain/discomfort	1	1	52 (85%)	81.54	−1
		2	9 (15%)	86.33	0
	2	2	45 (80%)	77.77	0
		3	10 (18%)	64.50	−1
		4	1 (2%)	50.00	0
Anxiety/depression	1	1	84 (94%)	81.38	−1
		2	5 (6%)	71.80	0
	2	2	23 (82%)	73.48	0
		3	4 (14%)	67.50	−1
		4	1 (4%)	60.00	0

*The size of inconsistency of ≤ 0 indicated consistency.

### Convergent validity

Table 4 demonstrates the Spearman’s correlation coefficients between the EQ-5D and SF-36v2 dimensions. In general, the pattern of correlations between the 2 versions of EQ-5D and SF-36v2 was similar. As expected, stronger correlation between similar dimensions of EQ-5D and SF-36v2 were found: mobility and physical functioning (r = 0.54 for the 3L, r = 0.53 for the 5L); pain/discomfort and bodily pain (r = 0.30 for the 3L, r = 0.35 for the 5L); anxiety/depression and mental health (r = 0.45 for the 3L, r = 0.49 for the 5L). However, self-care and usual activity dimension of the EQ-5D were weakly associated with various dimensions of SF-36v2. Additionally, Pearson’s correlation coefficient between the VAS score and index value was also similar between the 3L and 5L (0.36 for the 3L, 0.35 for the 5L with p-value < 0.001).

**Table 4:** **Correlation coefficients between EQ-5D and SF-36v2 dimensions**

**Dimension**	**PF**	**RP**	**BP**	**GH**	**VT**	**SF**	**RE**	**MH**
**3L**								
Mobility	.54**	.28**	.41**	.42**	.25**	−0.07	0.11	0.14
Self-care	0.16	0.05	.19*	0.12	0.14	0.16	0.06	0.18
Usual activities	.25**	.21*	.30**	.19*	.27**	0.18	0.13	.28**
Pain/discomfort	.19*	0.17	.30**	.24**	.18*	0.11	.21*	.22*
Anxiety/depression	0.05	0.09	.23*	.22*	.21*	.32**	.29**	.45**
**5L**								
Mobility	.53**	.29**	.44**	.44**	.23*	−0.08	0.09	0.11
Self-care	.24**	.20*	.23*	0.18	0.16	.24**	.21*	.22*
Usual activities	.30**	.23*	.29**	.22*	.24*	0.16	0.14	.24**
Pain/discomfort	.24**	.23*	.35**	.28**	.22*	0.08	0.16	0.18
Anxiety/depression	0.08	0.12	.19*	.21*	.28**	.35**	.29**	.49**

PF (physical functioning), RP (role limitation due to physical problems), BP (bodily pain), GH (general health perceptions), SF (social functioning), VT (vitality), RE (role limitations due to emotional problems), MH (general mental health).

*Correlation is significant at the 0.05 level (2-tailed).

**Correlation is significant at the 0.01 level (2-tailed).

### Discriminative power

The absolute informativity (*H* ′) of the 5L was higher than the 3L for all dimensions as shown in Table 5. This reflects that the 5L generated more informativity than the 3L. We also found that the 5L generated similar results compared with the 3L when it came to relative informativity (*J* ′).

**Table 5:** **Shannon’s index** (*H* ′) **and Shannon’s Evenness index** (*J* ′) **of 3L and 5L**

**Dimension**	***H*** **′**		***J*** **′**	
	**3L**	**5L**	**3L**	**5L**
Mobility	0.85	1.20	0.53	0.52
Self-care	0.12	0.21	0.08	0.09
Usual activities	0.70	0.78	0.44	0.34
Pain/discomfort	1.00	1.40	0.63	0.60
Anxiety/depression	0.79	1.06	0.50	0.46

### Test-retest reliability

The time interval between the first and second test was approximately 3 weeks. Overall, the reliability coefficient and percentage agreement of the 5L were slightly lower than the 3L (Table 6). The weighted kappa coefficient for the 3L ranged between 0.39 and 0.70, and between 0.44 and 0.57 for the 5L; this indicated that the 3L had better reproducibility than the 5L. The percentage agreement returned higher values than the weighted kappa coefficient; it was between 0.78 and 0.98 for the 3L and 0.67 and 0.97 for the 5L. The ICCs of the 3L and 5L indexes were 0.64 and 0.70, respectively, which indicated excellent reproducibility for both instruments.

**Table 6:** **Test-retest reliability of the 3L and the 5L**

**Dimension**	**Weighted kappa coefficient (95% CI)**		**Percentage agreement**	
	**3L**	**5L**	**3L**	**5L**
Mobility	0.70 (0.53-0.86)	0.57 (0.40-0.74)	0.89	0.83
Self-care	n/a*	n/a	0.98	0.97
Usual activities	0.39 (0.16-0.62)	0.45 (0.25-0.65)	0.82	0.81
Pain/discomfort	0.56 (0.39-0.72)	0.44 (0.29-0.58)	0.78	0.67
Anxiety/depression	0.50 (0.31-0.70)	0.49 (0.33-0.65)	0.82	0.77
	Intraclass correlation coefficient (ICC)**			
EQ-5D index	0.64 (0.51-0.74)	0.70 (0.57-0.79)		

*Not enough information to calculate kappa coefficient for self-care dimension.

**ICC was 2-way random, single measures, and absolute agreement.

### Patient preferences

Thirty-six percent of respondents indicated that the 5L was easier to answer than the 3L while 33% of respondents indicated that there was no difference between the 5L and the 3L. In terms of reflecting health status, most respondents (63%) agreed that the 5L was better in describing their health states while 29% indicated that both versions were similar.

## Discussion

This report is the first study in Thailand that assesses the measurement properties of the 5L and compares it with the 3L. Similar to previous studies [16,18,20,21,23,24], self-care showed the highest percentage of ceiling effect in both the 3L and 5L. On the other hand, the lowest ceiling was found in pain/discomfort (44%) [18,21,23]. Similar to the previous studies [16,18-21,23,24], the proportion of the ceiling in our study was lower in the 5L (29%) compared with the 3L (33%). However, in the previous studies that involved patients with a variety of severity higher reduction in ceiling of the 5L (3-17%) was identified [16,18,21,23]. The smaller reduction in ceiling found in our study is probably due to the fact that our respondents were likely to perceive that they were healthy, which was consistent with their median VAS score of 0.78. In fact, our finding is similar to those of the previous study [20], which found a slight reduction in ceiling effect among participants; whose median VAS score was 80.

In each dimension, more than half of the responses were in level 1 (no problem) for both the 3L and 5L. In addition, we found that the majority of level 1 in the 3L still remained at level 1 in the 5L (85-98%) while only 2% (self-care) to 15% (in pain/discomfort) were upgraded to level 2 in the 5L. The redistribution from 3L-level 2 (some problems) to 5L-level 2 (slight problems) was also high, ranging from 69% for mobility to 100% for self-care. On the other hand, redistribution from 3L-level 2 to 5L-level 3, ranging only from 9% for usual activities to 22% for mobility. This is probably due to the fact that most respondents in our study perceived that they were healthy and have no problem. In addition, for those who indicated having some problems in the 3L they are more likely to have slight problems rather than moderate problems. This finding supports that the 5L can present more details of severity than the 3L and that the inclusion of the slight problems (level 2) in the 5L is essential, especially when the respondents were in mild condition. However, no supportive evidence of the inclusion of severe problems (level 4) in the 5L was found in our study as no 3L-level 3 responses were reported. Again, this may also be due to the fact that our respondents were likely to perceive that they were healthy.

No inconsistent responses were found in our study. This indicates that our respondents were able to consistently answer both the 3L and 5L. This is similar to previous studies [15,18,20,21,23,24] which showed that inconsistency was quite low, ranging from 0.5% to 3.5%. However, the consistent responses may be due to the low number of the sample size and the characteristics of our sample - educated and healthy diabetic patients. In addition, even when the respondents completed the questionnaires themselves, they were well-advised by trained staff. However, it should be noted that the single page of the 3L and 5L response scale used in this study was against the standards for the EQ-5D which should be used separately in one page A4 format. As the result, the answers from the 3L and the 5L may not be totally independent and might generate less reliable results.

The measurement of reliability and agreement is important in health classification as it reveals the amount of errors of the measurement. The concept of ‘reliability’ differs from ‘agreement’ in that reliability is a relative measure which is the ratio of variability between subjects to the total variability of all measurement in the sample [36]. Thus, it reflects the ability of an instrument to differentiate between subjects. In contrast, an agreement is an absolute measure which is the degree to which responses are identical. Cohen’s weighted kappa is often used in assessing test-retest reliability of ordinal instruments as it takes the chance agreement into account. However, the lack of variance in the data set meant that the kappa could not be calculated so it was necessary to rely on the percentage agreement values. However, it should be cautioned that the percentage agreement may give higher reproducibility figures than the kappa coefficient [35].

Unlike previous studies [21,23,24], our results of the test-retest reliability/agreement showed that the 5L was slightly less reproducible than the 3L in all dimensions. This is probably due to the fact that the average time interval between the two tests was too long (approximately 14–21 days) so the condition of the patients might have changed [36]. If this is the case there is a higher chance of distorting the 5L results as the 5L is better than the 3L in capturing small changes in health status. In fact, a simple question such as “Has your health changed significantly since last time you filled in the questionnaire?” should be added and only patients whose conditions were stable should be included in the test-retest analysis. Since there is no check whether health status of the patients was changed or remained the same the result of test-retest reliability should be interpreted with cautions.

Convergent validity was evaluated by correlations between the EQ-5D and SF-36v2 dimensions. Both the 3L and 5L presented an acceptable degree of association and similar correlation pattern with the SF-36v2 in some pairs of dimension, i.e. mobility versus physical functioning; pain/discomfort versus bodily pain; and anxiety/depression versus mental health. The findings were similar to the study by Kimman et al. [28] that assessed the relationship of the 3L with the SF-36v2 among the occupational population in Thailand.

Similar to previous studies [15,16,20], absolute informativity (*H* ′) increased in all dimensions for the 5L while in terms of the evenness of distribution evaluated by Shannon’s Evenness index (*J* ′), the 5L was comparable to the 3L. While the maximum value of *H*′ for the 5L is 2.32, our *H*′ values ranged from 0.21 to 1.40 which was lower than the findings from Pickard et al. [16] (0.84-2.00) and Janssen et al. [15] (2.05-2.26). With the maximum value of *J* ′ set at 1.00, our *J* ′ values ranged from 0.09 to 0.60 which was also lower than Pickard et al. [16] (0.36-0.86) and Janssen et al. [15] (0.88-0.97). The lower *H* ′ and *J* ′ values found in our study may have risen from the mild characteristic of our sample since the extreme problems (3L-level 3 and 5L-level 5) were not reported. As the result, the levels of responses of the EQ-5D were used ineffectively, resulting in low *H* ′ and *J* ′ values.

In our study, diabetic mellitus was chosen as it is a common chronic disease that substantial affects quality of life [37,38]. Additionally, diabetes was ranked as third and eighth in terms of Disability Adjusted Life Year (DALY) loss in Thai women and men, respectively [39]. We included patients with no complications in our study to ensure that the health status will be stable enough in order to test the test-retest reliability/agreement. However, given the mild condition of our sample, we were unable to assess the redistribution of answers from the 3L-level 3 to the 5L.

Further studies should be conducted for patients with a variety of severe health problems. In addition, it should be noted that the generalizing of the findings to different groups of patients should be made with caution as the pattern of responses may differ by disease characteristics [8]. One further limitation is that the 5L index values were obtained from the interim mapping generated by the EuroQol group since the valuation study for the 5L in Thailand has not been completed yet. Although the calculation was based on the Thai 3L value sets, the results of the mapping may deviate compared to the actual responses [40]. In addition, it is also worth noting that about 20% of our respondents were in the age 12–15 years old. Although the use of adult version may be allowed among this age group of respondents there is very limited evidence on the suitable of the use of adult version especially in term of validity and reliability among this group of respondents.

## Conclusions

In summary, this study suggests that the 5L was greater than the 3L in terms of distribution, ceiling, informativity, discriminatory power, and patient preferences. The 5L also showed reasonable convergent validity and test-retest reliability. Thus, the 5L should be recommended for use in research or clinical practice and can also be used as a preferred health-related quality of life questionnaire in Thailand.
